# Mid-Infrared Spectroscopy as a Rapid Tool to Qualitatively Predict the Effects of Species, Regions and Roasting on the Nutritional Composition of Australian Acacia Seed Species

**DOI:** 10.3390/molecules26071879

**Published:** 2021-03-26

**Authors:** Oladipupo Q. Adiamo, Yasmina Sultanbawa, Daniel Cozzolino

**Affiliations:** 1 ARC Training Centre for Uniquely Australian Foods, Queensland Alliance for Agriculture and Food Innovation (QAAFI), The University of Queensland, Block 10, Level 1, 39 Kessels Rd., Coopers Plains, QLD 4108, Australia; o.adiamo@uq.net.au (O.Q.A.); y.sultanbawa@uq.edu.au (Y.S.); 2 Centre for Nutrition and Food Sciences, Queensland Alliance for Agriculture and Food Innovation (QAAFI), The University of Queensland, Brisbane, QLD 4072, Australia

**Keywords:** acacia seed, species, regions, roasting, MIR spectroscopy, chemical composition

## Abstract

In recent times, the popularity of adding value to under-utilized legumes have increased to enhance their use for human consumption. Acacia seed (AS) is an underutilized legume with over 40 edible species found in Australia. The study aimed to qualitatively characterize the chemical composition of 14 common edible AS species from 27 regions in Australia using mid-infrared (MIR) spectroscopy as a rapid tool. Raw and roasted (180 °C, 5, 7, and 9 min) AS flour were analysed using MIR spectroscopy. The wavenumbers (1045 cm^−1^, 1641 cm^−1^, and 2852–2926 cm^−1^) in the MIR spectra show the main components in the AS samples. Principal component analysis (PCA) of the MIR data displayed the clustering of samples according to species and roasting treatment. However, regional differences within the same AS species have less of an effect on the components, as shown in the PCA plot. Statistical analysis of absorbance at specific wavenumbers showed that roasting significantly (*p* < 0.05) reduced the compositions of some of the AS species. The results provided a foundation for hypothesizing the compositional similarity and/or differences among AS species before and after roasting.

## 1. Introduction

Acacia seed (also known as wattle seed) has been used as a food source by the Australian indigenous people for thousands of years [1]. Generally, acacia species are used in planting programs for the purpose of enriching degraded land [2]. There are over 960 species of Acacia seed (AS) in different regions in Australia, out of which 40 species were found to be edible [1,3,4,5]. The most common of the AS species is the Elegant wattle (*Acacia victoriae*) and the species is considered as the food industry standard [6]. AS is a good source of protein, dietary fibre, and minerals such as potassium, sodium, and iron [7]. The seeds are not consumed raw but are usually roasted mainly to develop a unique nutty flavour and aroma depending on the species and roasting conditions [7,8]. Moreover, roasting is carried out to reduce the anti-nutrients and protease inhibitors, particularly trypsin inhibitors in AS [9].

In recent times, there has been a gradual increase in the demand for AS as part of the commercial bush food market. The ground roasted *A. victoriae* seed have been used as ingredients in condiments and coffee analogue [10,11]. However, the seeds of other species have not been fully utilized as an ingredient in food products, probably due to limited scientific information on the species [10,11]. In order to identify the AS species with high nutritional and organoleptic qualities, compositional characterization of seeds of these species is essential [9,10,11]. Since there are several edible AS species, the use of conventional chemical analysis may be difficult due to its limitations such as time-consuming protocols, the use of toxic and expensive reagents, and the need for specialized personnel. Therefore, high-throughput analytical tools may be required to overcome these limitations.

Spectroscopy techniques are well-established techniques for determining the chemical components of foods due to its rapidity, simplicity, safety, and ability to measure multiple attributes simultaneously without the monotonous preparation of the sample [12,13,14]. Mid-infrared (MIR) spectroscopy has been used to determine the chemical composition in grains [15], fruits [16], meat [17], and other agricultural products. MIR spectroscopy checks the fundamental vibrational and rotational stretching of molecules, which gives the sample’s chemical profile [18]. The region (400 to 4000 cm^−1^) of MIR is a robust and reproducible part of the electromagnetic spectrum that can give reliable differences in sample compositions [19]. A review of the literature shows that there is no information on the use of MIR spectroscopy to predict the chemical composition of AS species. Therefore, the aim of the study was to use MIR spectroscopy as a rapid tool to qualitatively predict the chemical composition of raw and roasted Australian AS species from different regions.

## 2. Materials and Methods

### 2.1. Sample Preparation

Ten AS species (A. baileyana, A. calamifolia, A. longifolia spp longifolia, A. longifolia spp sophorae, A. microbotrya, A. obteca, A. pravissima, A. provincialis, and A. pycnantha) were purchased from Wattle Seeds Australia (Tarrington, Victoria, Australia). Additionally, three AS species (A. victoriae, A. coriacea, and A. cowleana) were purchased from NATIF Australian Superfoods, Fruits, Herbs, Spices and Mixes (Victoria, Australia). The last species (A. colei) were supplied by Environs Kimberley, Kimberley, Western Australia. The suppliers sourced the harvested AS species from different regions in Australia, as shown in Table 1. The raw seeds were divided into two sub-sets. The first set was roasted in a preheated oven at 180 °C at three different times (5, 7, and 9 min) and then ground to flour using a Retsch oscillating ball mill (Retsch NM400, 42,781 Haan, Germany). Both, raw and roasted AS flour samples were used for MIR analysis.

### 2.2. Spectra Collection

MIR spectroscopy with a platinum diamond attenuated total reflectance (ATR; Bruker Optics GmbH, Ettlingen, Germany) was used to collect the raw and roasted AS flour spectra. Flour samples were added on top of the ATR sample module where samples were scanned in the mid-infrared range from 400 to 4000 cm^−1^. The OPUS software version 8.5 provided by Bruker Optics (Bruker Optics GmbH) was used to record the MIR spectra. Thirty-two scans were obtained and co-added for each sample of flour at a resolution of 4 cm^−1^. A background spectrum (air) was obtained by collecting 32 scans following cleaning of the crystal with a mixture of ethanol and water (70% *v*/*v*).

### 2.3. Data Analysis

Spectra were exported in csv format into The Unscrambler (Camo, Norway) software for chemometric analysis. The spectra were pre-processed using Savistky–Golay second derivative (second polynomial order and 11 data smoothing points) [20]. Then, principal component analysis (PCA) was done using full cross-validation (one leave out) [21]. A statistical analysis of the means of absorbance values at specific wavenumbers was conducted using IBM SPSS Statistics 23 (SPSS Inc., USA). Differences in the means were compared using Duncan’s multiple range tests and significance was accepted at *p* < 0.05.

## 3. Results and Discussion

### 3.1. Essential Features in the MIR Spectra Related to the AS Samples

**Figure 1:**
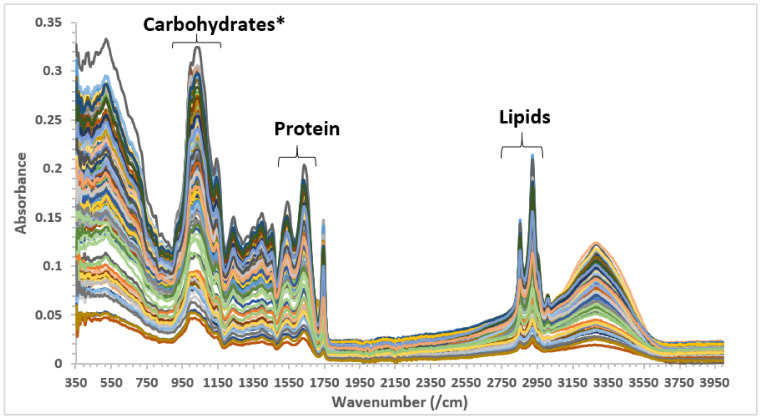
Mid-infrared spectra of raw and roasted acacia seed species collected from different regions in Australia. * Structural and non-structural carbohydrates.

The ATR-MIR spectra and the relevant MIR features found in the set of AS samples analyzed are presented in Figure 1. The MIR spectra of the samples displayed frequencies in the MIR spectra range related to structural and non-structural carbohydrate (900 to 1240 cm^−1^), proteins (1500 to 1700 cm^−1^), and lipids (2800 to 2950 cm^−1^) [18,22,23,24,25]. The MIR band region around 1744 cm^−1^ was reported to be associated with phosphodiesters [18,23,24,26]. In addition, the region between 900 and 1500 cm^−1^ is characterized by the presence of bands derived from the aromatic O-H bend, the aromatic C-H in plane bend, and the C-O stretch of phenolic compounds [27,28]. Moreover, the MIR spectra showed specific peaks associated with amide-I (C-O stretch, C-N stretch) around 1641 cm^−1^ and with starch around 1045 cm^−1^, or lipids and fatty acids (aliphatic CH_2_ groups) at 2852 and 2926 cm^−1^ [18,22,23,24,25,29].

### 3.2. Principal Component Analysis

**Figure 2:**
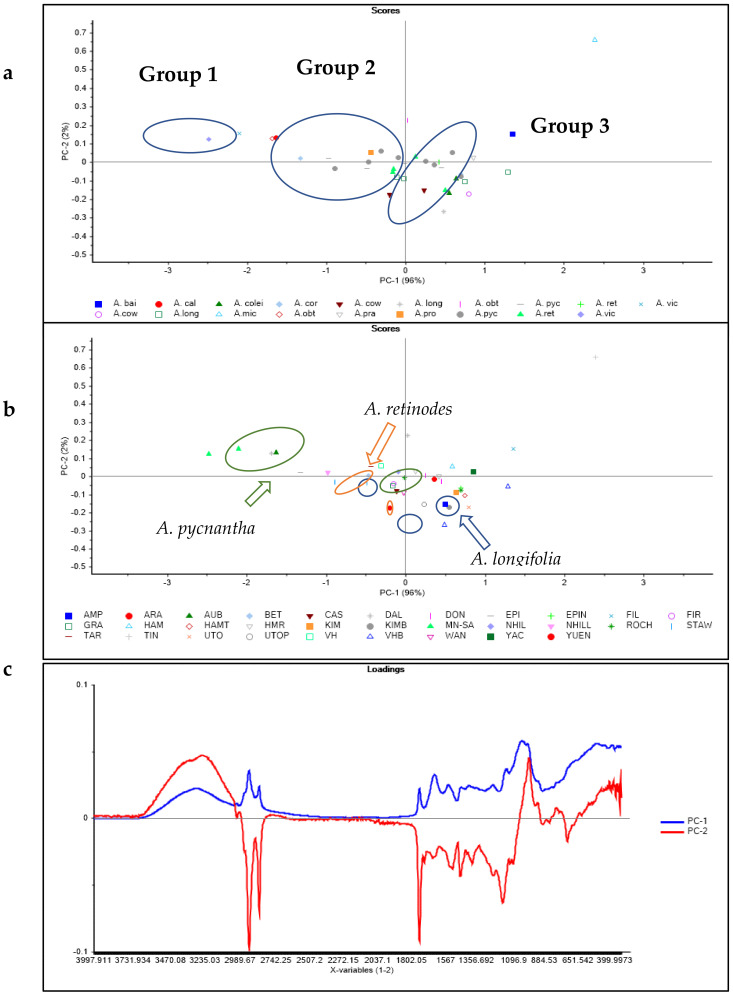
Principal component score plots of raw AS samples analysed by mid-infrared spectroscopy and labelled by (**a**) species, (**b**) region, and (**c**) their corresponding loadings.

The principal component (PC) score plot of the AS samples analyzed and labelled by species and region are shown in Figure 2a,b. Principal component 1 (PC1) accounts for 96% of the total variability (98%), which explains the variability in the set of samples analyzed. The species were clustered into three groups on the PC1 axis (Figure 2a). Group 1 consists of *A. victoriae*, *A. calamifolia*, and *A. obteca*, which are found in the negative axis of PC1. Additionally, the following species: *A. baileyana*, *A. pravissima*, and *A. colei* made up Group 2 and were clustered in the positive axis of PC1. Although the remaining species are found in Group 3 (clustered at the middle of PC1), there are some species (*A. pycnantha*, *A. longifolia*, and *A. coriacea*) that occur in both Group 2 and 3 (Figure 2a). The species that are grouped together have common compositions and can be evaluated together.

Figure 2b shows the separation and clustering of the AS samples according to region. Comparing the regions within the same species, for instance in *A. longifolia* species, it could be observed that the seeds harvested from CAS and WAN regions were separated from those obtained from VHB. Similarly, there was clustering of *A. pycnantha* samples obtained from NHILL, STAW, and VH at the negative axis of PC1 while those obtained from DON, ARA, and HAM were found in the positive PC1 axis. However, regions have less of an effect on the compositions of *A. retinodes* samples obtained from different regions. *Acacia retinodes* samples harvested from GRA, FIR and HMR were found to cluster at the origin of the PC plot (Figure 2b).

Figure 2c shows the loading plot derived from the PCA analysis of the AS samples. It can be interpreted from the loadings that frequencies associated with aliphatic CH_2_ groups (e.g., lipids), N-H (e.g., proteins, amino acids), and C-H (polysaccharides) might explain the observed differences between the AS species and regions. Furthermore, the differences in the samples within the same species may be due to variation in the soil compositions and environmental conditions between regions.

**Figure 3:**
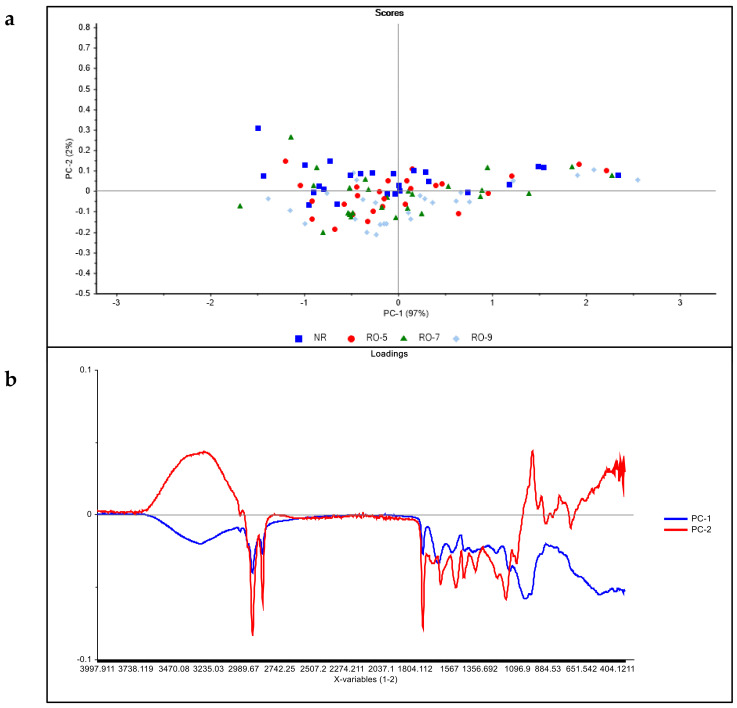
Principal component score plot (**a**) and loadings (**b**) of raw and roasted AS samples analysed by MIR spectroscopy.

The effect of the roasting period on the separation of the AS samples on the PC plot (Figure 3a) clearly shows that the raw samples were grouped on the positive side of PC2. However, two raw samples were found in the negative axis of PC2, which may be due to variation in the composition of some of the AS species. On the other hand, samples roasted for 5 and 7 min were evenly distributed in the PC2 plot. Further roasting of the seeds up to 9 min resulted in the clustering of the roasted (9 min) samples in the negative axis of PC2 (Figure 3a). The differences between the non-roasted and roasted samples may be attributed to the removal of moisture from the seed flour, thereby allowing the amounts of other components to increase. In addition, roasting may result in the formation of new compounds or the release of bound compounds such as phenolic and non-phenolic compounds with potential antioxidant properties [30,31,32]. Moreover, previous studies have demonstrated that a unique aroma and flavor was perceived in five AS species after roasting [8] and the aroma and flavor compounds may have been formed or released during roasting. However, the impact of roasting (180 °C) on AS may vary among the AS species due to the differences in their sizes and dimension. The order of size and dimensions are as follows: *A. obteca* > *A. coriacea* > *A. pravissima > victoriae* > other species of similar size. Heat will distribute faster in small-sized seeds than bigger-sized seeds and this could be responsible for the findings in the roasted samples (Figure 3).

Figure 3b shows the loading plot derived from the PCA analysis of the raw and roasted AS samples. Similarly, the frequencies associated with aliphatic CH_2_ groups (e.g., lipids), N-H (e.g., proteins, amino acids) and C-H (polysaccharides) may be responsible for the differences between the raw and roasted AS samples. In addition, frequencies associated with aromatic and flavonoid groups might also explain the separation of the raw and roasted AS samples.

### 3.3. Analysis of Absorbance at Specific Wavenumbers

**Figure 4:**
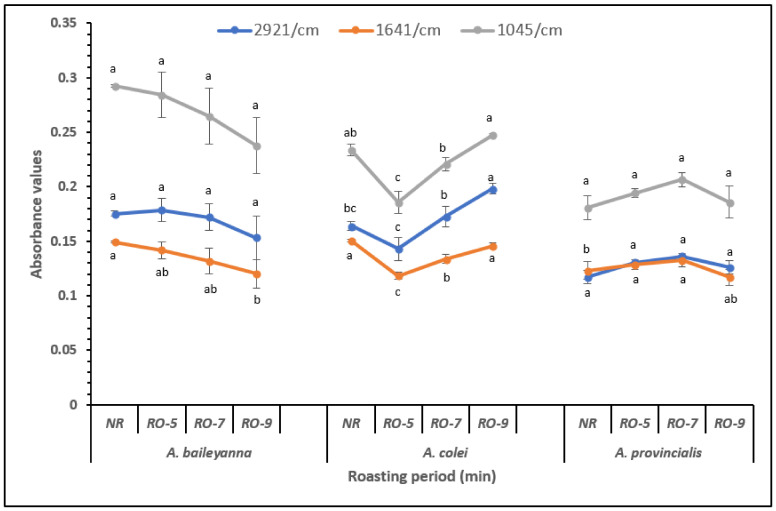
Absorbance values at specific wavenumbers derived from the analysis of acacia seed samples at different roasting periods (min) using mid-infrared spectroscopy. Means with the same letter are not significantly different (*p* < 0.05).

Further analysis of the MIR data was carried out by plotting the absorbance values at specific wavenumbers in order to observe any changes related to roasting treatment. Three AS species (*A. baileyana*, *A. colei*, and *A. provincialis*) were significantly affected by roasting, and this was presented in Figure 4. The absorbance values at 1045 cm^−1^, 1640 cm^−1^, and 2921 cm^−1^ associated with starch, amide I, and lipids, respectively, of *A. colei* sample were reduced (*p <* 0.05) after roasting for 5 min. However, further roasting of the seed up to 9 min raised the absorbance values but the values were not significantly (*p >* 0.05) different from the raw samples. On the other hand, the absorbance values at 1640 cm^−1^ (e.g., associated with protein) of *A. baileyana* were found to decrease after roasting for 9 min. Unlike the last two species mentioned, roasting of *A. provincialis* for 5 and 7 min raised (*p <* 0.05) the absorbance values at 1640 cm^−1^. Similar observations were reported in roasted coffee analyzed using infrared spectroscopy by other authors [33]. Heat treatment may result in the unfolding of the polypeptide structure of protein, which might then interact with the starch component through the formation of Maillard reactions, resulting in the reduction of starch and protein availability and content [31]. Moreover, Maillard products and volatile compounds may be formed during roasting [34]; therefore, qualitative and quantitative studies should be conducted on the products and compounds formed after roasting as this may influence the ingredient composition and type of products to be developed with AS. Prolonged roasting of the seed may cause further unfolding of the polypeptide chain which may result in the intramolecular binding of the polypeptide, protein aggregation, and less interaction with other components [9,29,30,31,34]. Furthermore, heat treatment may cause protein denaturation, as observed in the variation in the amide I region [35,36]. Denaturation may influence the solubility and functional properties of protein depending on the degree of denaturation [37,38]. Therefore, further studies should be conducted to determine the effect of the roasting period on the functional properties of the affected AS species.

## 4. Conclusions

This study showed for the first time the utilization of MIR spectroscopy as a promising tool to qualitatively predict the differences and similarities in the composition of edible AS species from different regions. The PCA analysis showed that the differences in the samples are mainly influenced by species and the duration of roasting treatments. Compared within AS species, regions have less of an influence on the variation in the spectra of the species, as observed in the PCA plot. The loadings derived from the PCA analysis highlighted specific wavenumbers in the MIR associated with the composition of the samples. The application of this technique will offer the possibility to develop calibrations between the MIR spectra and the reference methods in order to measure several parameters simultaneously, thereby reducing the time and cost of analysis. The incorporation of these rapid and low-cost analytical tools to both measure the composition and monitor the processing might be implemented throughout the AS value chain, providing huge benefits to this industry. Further studies will be carried out by using MIR spectroscopy to develop calibrations to quantitatively predict the chemical composition of the raw and roasted AS samples.

## Figures and Tables

**Table 1:** The Australian acacia species used in this study showing their regions and harvest date.

Species (spp. Abbreviation *)	Common Name	Regions	Abbreviation **	Harvest Date
*Acacia baileyana (A.bai)*	Cootamundra wattle	Firgrove bulk, South West (SW) Victoria	FIL	December 2019
*Acacia calamifolia (A. cal)*	Wallowa wattle	Auburn, South Australia (SA)	AUB	December 2019
*Acacia colei (A. col)*	Cole’s wattle	Kimberly, Western Australia (WA)	KIM	December 2019
*Acacia coriacea (A. cor)*	Wiry wattle	Epinara station, North East (NE) Alice Springs	EPI	November 2018
*Acacia cowleana (A. cow)*	Halls creek wattle	Utopia, NE Alice Springs	UTO	November 2018
		Yuendumu, North West (NW) Alice Springs	YUEN	November 2018
*Acacia longifolia* *spp. longifolia (A. lon)*	Coastal wattle	Hamilton/Firgrove	HAMT	December 2019
		Wannon, SW Victoria	WAN	December 2019
		Casterton (Schurmanns), SW Victoria	CAS	December 2019
*Acacia longifolia* spp. sophorae (*A. long*)	Coastal wattle	Victor Harbour, SA coast	VHB	December 2019
*Acacia microbotrya (A. mic)*	Manna wattle	Tincurren, WA	TIN	December 2019
*Acacia retinodes (A. ret)*	Swamp wattle	Harmans road, SW, Victoria	HMR	January 2020
		Firgrove sein Bulk, SW Victoria	FIR	December 2019
		Amphitheatre, SW Victoria	AMP	December 2019
		Grampians, SW Victoria	GRA	December 2019
*Acacia pravissima (A. pra)*	Ovens wattle	Yackandandah, NE Victoria	YAC	December 2019
*Acacia provincialis (A. pro)*	Water/swamp wattle	Tarrington, SW Victoria	TAR	December 2018
*Acacia pycnantha (A. pyc)*	Golden wattle	Rochester, NE Victoria	ROCH	December 2019
		Nhill, NW Victoria	NHILL	December 2019
		Donald, NW Victoria	DON	December 2019
		Stawell, NW Victoria	STAW	December 2019
		Ararat, SW Victoria	ARA	December 2019
		Betley, Central Victoria	BET	December 2019
		Victor Harbour, SA coast	VH	December 2019
		Hamilton/Wycliffe Seins bulk, SW Victoria	HAM	December 2019
*Acacia obteca (A. obt)*		Dalwallinu, WA	DAL	December 2019
*Acacia victoriae (A. vic)*	Elegant wattle	Mid-north, SA	MN-SA	December 2019

* abbreviations for species, ** abbreviations for regions.

## Data Availability

The data presented in this study are available on request from the corresponding author.

## Sample Availability

Samples of the compounds are available from the authors.
